# Acute Pancreatitis in a Previously Exocrine Pancreatic Insufficient Cystic Fibrosis Patient Who Had Improved Pancreatic Function After Being Treated With Lumacaftor/Ivacaftor

**DOI:** 10.1097/PG9.0000000000000096

**Published:** 2021-07-12

**Authors:** Alexander W. Redman, Michelle Yoo, Peter Freswick, Karen Thompson

**Affiliations:** From the * Internal Medicine/Pediatrics Residency, Spectrum Health/Michigan State University, Grand Rapids, MI; † Pediatrics Residency, Spectrum Health/Michigan State University, Grand Rapids, MI; ‡ Pediatric Gastroenterology, Helen DeVos Children’s Hospital, Grand Rapids, MI; § Pediatric Pulmonology, Helen DeVos Children’s Hospital, Grand Rapids, MI.

**Keywords:** pancreatitis, acute pancreatitis, cystic fibrosis, Orkambi, highly effective modulator therapy, ivacaftor, lumacaftor, elexacaftor, tezacaftor, pancreatic insufficiency, exocrine pancreatic insufficiency, CFTR, CFTR modulator therapy, pancreatic enzyme replacement therapy

## Abstract

Exocrine pancreatic insufficiency (EPI) is a common complication of cystic fibrosis (CF). While previously considered to be irreversible, recent reported cases document improved pancreatic function in CF patients with mild mutations after ivacaftor treatment alone. We report a 12-year-old female with homozygous F508del CF and EPI who developed acute pancreatitis after 3 years on lumacaftor/ivacaftor and subsequently had improved pancreatic function. As CF therapies advance, some EPI CF patients with more severe CF transmembrane conductance regulator mutations may see improved pancreatic function and subsequently develop pancreatitis.

## INTRODUCTION

Cystic fibrosis (CF) is a progressive genetic disease caused by dysfunction of CF transmembrane conductance regulator (CFTR) associated with significant morbidity and multisystemic complications. In the pancreas, CFTR is essential for ductal fluid production and bicarbonate secretion.^1^ While the reduction in function can be variable based on the severity of the respective CFTR mutation, many CF patients will develop exocrine pancreatic insufficiency (EPI). EPI was previously considered to be irreversible, and CF patients with EPI were thought to require lifelong pancreatic enzyme replacement therapy (PERT).

There have been significant advancements in CF treatments in recent years stemming from the use of CFTR modulator medications. The effects of these medications can be seen across a multitude of clinical domains, including reports of CF patients with EPI who have shown improved pancreatic function following CFTR modulator therapy. In this report, we present the case of a 12-year-old female with CF and EPI who presented with acute pancreatitis and was found to have improved fecal elastase after being treated with CFTR modulator therapy.

## CASE REPORT

A 12-year-old female with CF (homozygous F508del) presented with 3 days of severe epigastric abdominal pain. She has a history of EPI (fecal pancreatic elastase-1 result of <15 µg/g at 16 days of age), gastroesophageal reflux disease, and CF-related liver disease (on ursodiol with normal liver enzymes, without cirrhosis or portal hypertension). When she was 9 years old, the patient was started on lumacaftor/ivacaftor (100–125 mg, 2 tabs twice daily). At age 12 years, she was transitioned to 200–125 mg, 2 tabs twice daily.

Her initial mild abdominal pain progressed quickly to severe epigastric pain and reduced appetite 3 days before her admission. The patient denied any recent illness or fevers, and the remainder of the review of systems was unremarkable. Evaluation on the night before presentation for the same complaint revealed elevated serum lipase level (557 U/L; normal <160 U/L). Right upper quadrant ultrasound obtained at that time was normal. Repeat lipase on admission showed an increase to 932 U/L. Given her persistent pain and increasing lipase level, she was diagnosed with acute pancreatitis and admitted for IV hydration and pain control. Repeat right upper quadrant ultrasound showed the pancreas had enlarged and become more echogenic compared with the previous ultrasound, consistent with acute pancreatitis.

Initially, due to her documented history of EPI, pancreatitis was not high on our differential diagnosis. Repeat fecal pancreatic elastase-1 level during admission was 318 µg/g (normal > 200, moderate to slight EPI 100–200, and severe EPI < 100). She was able to advance her diet, and by hospital day 2, her lipase had decreased to 72 U/L. She was discharged the following day with decreased pancreatic enzyme supplementation (pancrelipase) by 20% and to monitor for symptoms. However, due to poor weight gain, pancrelipase was returned to full dose 18 months after her pancreatitis episode. There were no repeat measures of pancreatic function performed at this time.

## DISCUSSION

F508del is the most common mutation in CF patients and causes severe misfolding of CFTR resulting in processing and trafficking defects. A retrospective study^2^ observed that “moderate–severe CFTR genotypes” such as F508del have significantly decreased risk of pancreatitis due to the development of EPI at an early age. Before the advent of modulator therapy, pancreatitis in CF was generally believed to occur almost exclusively in pancreatic sufficient patients.^3^

A recent report discussed EPI-labeled CF infants who had a spontaneous increase in their fecal pancreatic elastase-1; however, this has only been documented in CF patients having at least one CFTR gene mutation conferring residual function.^4^ We did not find any cases of spontaneous pancreatic function improvement in patients such as our patient with homozygous CF-causing mutations (classes I, II, or III).

Highly effective modulatory therapies (HEMT) (such as ivacaftor in heterozygote G551D CF patients) have been shown to improve pancreatic function.^1^ However, our case demonstrates the possibility of lumacaftor/ivacaftor in a homozygous F508del patient, a non-HEMT, improving pancreatic function in an adolescent child with subsequent development of pancreatitis.

One other case is reported of a similar pediatric patient with homozygous F508del mutation, previously diagnosed with EPI, who presented with acute pancreatitis after treatment with lumacaftor/ivacaftor.^5^ The etiology was unclear in this case, however, as follow-up pancreatic function testing was not documented.

The mechanism believed to be responsible for acute pancreatitis in CF is related to a balance between pancreatic acinar reserve and ductal obstruction.^3^ As CFTR dysfunction worsens, so does the severity of the ductal obstructions and pancreatic acinar reserve.

Reversing CFTR dysfunction with modulator therapy could lead to a “critical mass” of improved functional pancreatic acinar tissue that could lead to symptomatic pancreatitis in the presence of persistent ductal injury. We hypothesize our patient’s treatment with lumacaftor/ivacaftor and subsequent improved exocrine pancreatic function in the setting of persistent ductal injury may have led to her developing pancreatitis.

An open label trial that observed homozygous F508del EPI CF patients (fecal elastase-1 concentrations of <200 μg/g at baseline) aged 2–5 years treated with lumacaftor/ivacaftor for 24 weeks reported temporary improvement in the mean fecal elastase-1 concentration.^6^ This study demonstrated improvement of the fecal elastase-1 over the 24-week treatment (mean of 13.1 increased to mean 66.3, *P* = 0.0012), yet only 6 subjects ever had a fecal elastase-1 >200 mg/g (2 of whom saw improvement after cessation of lumacaftor/ivacaftor). These observations support our hypothesis regarding the pathological mechanism for our patient’s presentation.

However, we also must note that the extreme variability seen in fecal elastase-1 response among these patients to lumacaftor/ivacaftor, as well as our own patient’s unique clinical scenario, suggests just a small subset of CF EPI patients exists with enough residual acinar cell activity to demonstrate improved pancreatic function with modulatory therapy. Our patient’s subsequent resumption of full-dose PERT (if return to EPI status was indeed the etiology of her poor weight gain) could also indicate that this improvement was merely temporary, or possibly that recurrent pancreatitis caused a progressive decline in pancreatic exocrine function following her hospitalization.

With the emergence of both HEMT and non-HEMT, we may expect to see a subset of EPI CF patients presenting with improved pancreatic function and thus possible subsequent acute pancreatitis. This increased incidence could create a role for routine pancreatic function monitoring in patients on modulators. However, further research is needed to determine the utility of serial pancreatic function monitoring and biochemical markers that may predict which patients may develop improved pancreatic function.

Regardless, clinicians should realize that some historically EPI CF patients on modulatory therapy may have improved pancreatic function and subsequent increased risk of pancreatitis.

## ACKNOWLEDGMENTS

All authors contributed to the literature review, original manuscript, and editing of the final draft.
